# Supplementary material for: Potential of FDG-PET as Prognostic Significance after anti-PD-1 Antibody against Patients with Previously Treated Non-Small Cell Lung Cancer
Source: J Clin Med. 2020 Mar 7;9(3):725. doi: 10.3390/jcm9030725 (PMC7141299; doi:10.3390/jcm9030725)
Supplement: Supplementary file 1 [file jcm-09-00725-s001.pdf]

Table S1. **Incidence of irAEs according to the measurement of FDG uptake and TB**

**All patients** (n=85)	**Any irAEs** Yes / No (39 / 46)	***p*-value**	**Grade 3 or 4 irAEs** Yes / No (9 / 76)	***p*-value**
**Gender**				
Male (n=65)	33 / 32	0.12	6 / 59	0.43
Female (n=20)	6 / 14		3 / 17	
**TLG**				
High (n=59)	22 / 37	**0.02**	4 / 55	0.12
Low (n=26)	17 / 9		5 / 21	
**MTV**				
High (n=58)	22 / 36	**0.01**	5 / 53	0.44
Low (n=27)	17 / 10		4 / 23	
**SUV_{max}**				
High (n=52)	21 / 31	**<0.01**	5 / 47	0.27
Low (n=33)	18 / 15		4 / 29	
**TB**				
High (n=13)	7 / 5	0.53	2 / 11	0.62
Low (n=72)	30 / 42		7 / 65	

Abbreviations: TLG, total lesion glycolysis; MTV, metabolic tumor volume; SUV_{max}, the maximum of standardized uptake value; TB, tumor burden; irAEs, immune-related adverse events.
